# Occurrence of *Escherichia coli* carrying Shiga toxin-producing genes in buffaloes on smallholdings in Bangladesh

**DOI:** 10.14202/vetworld.2018.1454-1458

**Published:** 2018-10-19

**Authors:** Mukta Das Gupta, Arup Sen, Ashutosh Das

**Affiliations:** 1 Department of Microbiology and Veterinary Public Health, Faculty of Veterinary Medicine, Chittagong Veterinary and Animal Sciences University, Khulshi, Chittagong 4225, Bangladesh; 2 Department of Genetics and Animal Breeding, Faculty of Veterinary Medicine, Chittagong Veterinary and Animal Sciences University, Khulshi, Chittagong 4225, Bangladesh

**Keywords:** antimicrobial resistance, buffalo, Shiga toxin-producing *Escherichia coli*

## Abstract

**Background and Aim:**

Shiga toxin-producing *Escherichia coli* (STEC) has emerged as significant foodborne pathogens. Ruminants are the primary reservoir of the zoonotic STEC. In Bangladesh, previous studies reported the presence of STEC in cattle, goat, and sheep; however, there is little information about STEC carriage by buffaloes. This study aimed to determine the occurrence of STEC in healthy (absence of clinical signs and symptoms) buffaloes on smallholdings in Bangladesh and to assess the antimicrobial resistance pattern of identified STEC isolates.

**Materials and Methods:**

A total of 100 rectal swab samples were obtained from randomly selected buffaloes on 40 smallholdings in Chittagong Division, Bangladesh. Samples were subjected to bacteriological screening to identify *E. coli*. All *E. coli* isolates were examined for the presence of the Shiga toxin-producing genes - Shiga toxin 1 (*stx1*) and Shiga toxin 2 (*stx2*) using polymerase chain reaction. The antimicrobial susceptibility of identified STEC isolates was tested using the disk diffusion method.

**Results:**

Results show that 71 fecal samples were positive for *E. coli* in bacteriological screening. The proportion of buffaloes harboring STEC isolates was 11% (11/100) (95% confidence interval [CI] 6.1-18.8], of which 7% (7/100) (95% CI 3.2-13.9) and 4% (4/100) (95% CI 1.2-10.2) carried *stx1* and *stx2* genes, respectively. Antibiogram revealed that 91% (10/11), 73% (8/11), 55% (6/11), and 55% (6/11) STEC isolates were resistant to tetracycline, sulfamethoxazole-trimethoprim, erythromycin, and ampicillin, respectively. In contrast, 91% (10/11) STEC isolates were sensitive to ciprofloxacin, chloramphenicol, and gentamicin, whereas 73% (8/11) isolates were sensitive to ceftriaxone.

**Conclusion:**

This study highlights, for the first time, a significant proportion of fecal samples from healthy buffaloes on smallholdings in Bangladesh harboring antimicrobial-resistant STEC. Transmission of antimicrobial-resistant STEC from buffaloes to humans could pose an added risk to public health in rural Bangladesh.

## Introduction

*Escherichia coli* is primarily harmless gut flora in warm-blooded animals. However, some *E. coli* serotypes may cause foodborne illness in humans [1]. Among the toxigenic serotypes, Shiga toxin-producing *E. coli* (STEC) is the most significant one in public health perspective. A number of serotypes produce Shiga toxins, and these include enterohemorrhagic *E. coli*, enterotoxigenic *E. coli*, enteroaggregative *E. coli*, enteropathogenic *E. coli*, and enteroinvasive *E. coli* [2]. The primary virulence factor of STEC is called verotoxin, Shiga-like toxin, or Shiga toxin. There are two distinct types of Shiga toxins produced by STEC: Shiga toxin 1 (*stx1*) and Shiga toxin 2 (*stx2*) [3]. Shiga toxins cause the host cellular death by suppressing the protein synthesis [4], which, in turn, manifested by mild gastroenteritis to life-threatening hemolytic uremic syndrome in human.

STEC infection in human is primarily foodborne. The food product from the reservoir animals and water contaminated by their feces can pose the high risk of contamination with STEC either with their feces or intestinal contents [5,6]. Meat products such as sausage, minced meat, luncheon, and hamburger and also dairy products are mainly responsible for high-risk transmission of STEC. Cattle are recognized as the natural reservoirs of STEC; however, other ruminants are also important [7].

Buffaloes, as a reservoir of STEC, have been reported in many countries in Asia, Europe, and South America [8-10]. In Bangladesh, the occurrence of antimicrobial-resistant STEC in healthy cattle, sheep, and goat on smallholdings has been described previously [11-13]; despite 38% buffalo sampled after the slaughter in an urban area of Bangladesh found STEC positive [14], buffaloes on smallholdings in rural Bangladesh remain unscreened for the presence of STEC.

The present study aimed to assess the presence and the antimicrobial resistance pattern of STEC in healthy buffaloes on smallholdings in Bangladesh.

## Materials and Methods

### Ethical approval

Ethical approval for the study was granted by the Institutional Animal Ethics Committee, Faculty of Veterinary Medicine, Chittagong Veterinary and Animal Sciences University, Chittagong 4225, Bangladesh.

### Study area

The study was conducted between July 2015 and June 2016, in the coastal area in Chittagong Division, Bangladesh.

### Collection of samples

In total, 100 buffaloes on 40 smallholdings were sampled for the study. From each animal, fecal materials were collected by placing a sterile swab into recto-anal junction. The collected swab then dipped into a tube (5 ml) containing buffered peptone water (Oxoid, UK). Sample tubes were placed in ice-box until they shipped to the laboratory at the Microbiology and Veterinary Public Health Department, Chittagong Veterinary and Animal Sciences University. Demographic data (age, sex, and health status) on the animals sampled were also recorded. Sampled animals were divided into six subclasses - adult (≥1 year) and calf (<1 year); male and female; animal with poor farm hygiene; and animals with good farm hygiene.

### Isolation and identification of E. coli

For enrichment, samples in buffered peptone water were incubated at 37°C for 24 h in an incubator. A loopful from each of the overnight bacterial broth was then inoculated onto MacConkey agar (Oxoid, UK) and incubated at 37°C for 24 h. Pink-colored colonies on MacConkey agar were suspected as *E. coli* colonies. Smears from the suspected colonies were examined microscopically to detect Gram-negative rods using gram staining method. Colonies positive for Gram-negative rods were then streaked onto Eosin Methylene Blue (EMB) Agar (Oxoid, UK) and presumptively identified *E. coli* by observing characteristic green colonies with metallic sheen. The presumptive isolates were subcultured into tryptic soy broth (TSB) at 37°C for 24 h. Then, TSB cultures for each presumptive isolate were preserved by adding 15% glycerin and stored at −80°C for further use.

### DNA extraction

Total DNA was extracted using boiling method [15]. Briefly, a loopful of overnight *E. coli* colonies from 5% bovine blood agar was transferred into the 1.5 ml Eppendorf tube and mixed in 200 μl of deionized water. The mixture was vortexed and heated in boiling water for 5 min followed by centrifugation at 10,000 rpm for 5 min. The supernatant was collected as a DNA template and stored at −20°C until use.

### Detection of Shiga toxin-producing genes

To detect the Shiga toxin-producing genes in *E. coli* isolates, uniplex polymerase chain reaction (PCR) was performed using specific primers for *stx1* and *stx2* genes (Table-1) [16,17]. Each PCR reaction was run with a final volume of 50 μl containing 1 μl (each) primer (20 pmol), 1 μl of dNTPs, 0.2 μl Dream Taq DNA polymerase, 5 μl of 20 mM magnesium chloride, 40.8 μl molecular grade water, and 1 μl DNA template. PCR was performed using a Thermocycler (2720 Thermal cycler, Applied Biosystems, USA) with an initial DNA denaturation at 95°C for 3 min followed by 30-cycle amplification at 94°C for 30 s (denaturation), 58°C for 40 s (primer annealing), and 72°C for 1 min (DNA synthesis). The PCR products were electrophoresed on a 1.5% agarose gel stained with 0.5 μg/ml ethidium bromide (Sigma-Aldrich, USA). The sizes of PCR products in comparison with a 1 kb ladder (Thermo Scientific Fermentas) were inspected using an ultraviolet transilluminator (BDA digital, Biometra GmbH, Germany). A STEC strain isolated by Das Gupta *et al*. [11] was used as a positive control in gel electrophoresis.

**Table-1:** Primers used to detect Shiga toxin-producing genes, *st×1* and *st×2*.

Target gene	Primer sequence	Annealing temperature (°C)	Size of product (bp)	Reference
*st×1*	F: ACA CTG GAT GAT CTC AGT GG R: CTG AAT CCC CCT CCA TTA TG	58	~614	[16]
*st×2*	F: CCA TGA CAA CGG ACA GCA GTT R: CCT GTC AAC TGA GCA GCA CTT T	58	~779	[17]

### Antimicrobial susceptibility testing

Antimicrobial susceptibility of the STEC isolates was investigated using the disk diffusion method [18]. Commercial discs for ten commonly used antimicrobial agents in Bangladesh were used: Amoxicillin (AML, 10 μg), ampicillin (AMP, 10 μg), ceftriaxone (CRO, 30 μg), chloramphenicol (CHL, 30 μg), ciprofloxacin (CIP, 5 μg), doxycycline (DOX, 30 μg), erythromycin (E, 15 μg), gentamicin (CN, 10 μg), tetracycline (TE, 30 μg), and trimethoprim/sulfamethoxazole (SXT, 30 μg). The susceptibility pattern was denoted as “resistant “ and “sensitive” based on inhibitory zone and comparing with the standard from the Clinical and Laboratory Standards Institute [19].

### Statistical analysis

All data were entered into a spreadsheet program (Excel 2007, Microsoft Corporation). The proportion of animals carrying STEC with 95% confidence interval (CI) and significance of the difference between variables for the presence of *stx1* and *stx2* genes were calculated by Fisher’s exact test using GraphPad statistical software [20].

## Results

### Cultural characteristics of E. coli

Of the 100 fecal samples cultured in this study, 71 produced bright pink colonies on MacConkey agar plates and found as Gram-negative rods with gram staining. All 71 suspected isolates produced characteristic green colonies with the metallic sheen on EMB agar.

### Proportion of animal carrying STEC

**Figure-1:**
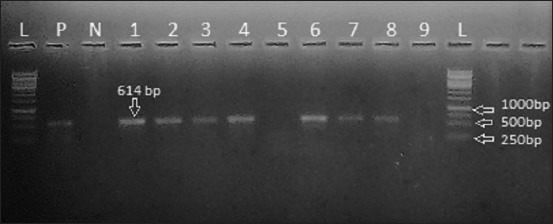
Agarose gel electrophoresis of PCR products amplified for Shiga toxin 1 (*stx1*) gene: Lanes (L): DNA ladder (1kb). Lane (P): Positive control. Lane (N): Negative control. Lanes 1-4 and 6-8 display positive Shiga toxin-producing *Escherichia coli* isolates that showed specific bands for *stx1* gene at 614 bp.

**Figure-2:**
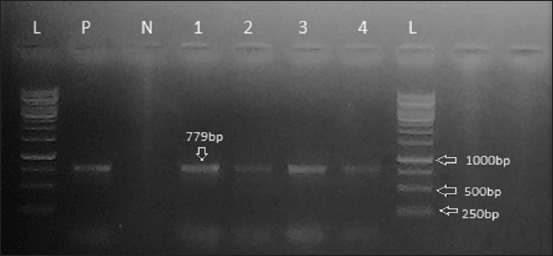
Agarose gel electrophoresis of PCR products amplified for Shiga toxin 2 (*stx2*) gene: Lanes (L): DNA ladder (1kb). Lane (P): Positive control. Lane (N): Negative control. Lanes 1-4 display positive Shiga toxin-producing *Escherichia coli* isolates that showed specific bands for *stx2* gene at 779 bp.

PCR results show that seven isolates were positive for the *stx1* gene (Figure-1) and four for the *stx2* (Figure-2), and hence, the proportion of animal harboring *E. coli* carrying *stx1* and *stx2* genes was 7% (95% CI 3.2-14.0) and 4% (95% CI 1.2-10.2), respectively. In total, 11% (95% CI 6.1-18.8) buffaloes were positive for STEC. The distribution of STEC in different categories of buffaloes sampled is presented in Table-2. Two calves and nine adult buffaloes were positive for STEC (p=0.323). The isolation rate of STEC from buffaloes with poor farm hygiene was significantly higher compared to buffaloes with good farm hygiene (Table-2).

**Table-2:** Distribution of Shiga toxin-producing genes, *st×1* and *st×2*, in *Escherichia coli* isolated from Buffaloes in Bangladesh.

Distributed by	Category of animals (n)	PCR positive for targeted genes		Total number positive for STEC	Proportion of animal carrying STEC (95% CI)	p-value

		*st×1*	*st×2*			
Age	Adult (66)	6	3	9	13.6 (7.1-24.1)	0.323
	Calves (34)	1	1	2	5.9 (0.6-20.1)	
Sex	Male (50)	4	1	5	10.0 (3.9-21.8)	1.000
	Female (50)	3	3	6	12.0 (5.3-24.1)	
Farm hygiene	Poor (31)	5	3	8	25.8 (13.5-43.5)	0.003
	Good (69)	2	1	3	4.4 (1.0-12.5)	

N=Number of animals sampled, STEC=Shiga toxin-producing *Escherichia coli*, CI=Confidence interval, PCR=Polymerase chain reaction

### Antimicrobial resistance pattern

Antimicrobial susceptibility test of 11 STEC isolates shows that 91% of the STEC isolates were resistant to TE, 73% to SXT, 55% to E, 55% to AMP, 45% to AML, 45% to DOX, and 27% to CRO (Table-3). Results also show 91% of STEC isolates to be sensitive to CHL, 91% to CIP, and 91% to CN. Eight (73%) STEC isolates showed multidrug resistance (MDR) as they were resistant to more than one antimicrobial agent. Results revealed seven different resistant patterns among STEC isolates (Table-3).

**Table-3:** Resistance pattern of STEC isolated from buffaloes in Bangladesh.

Number of isolates	Virulent genes		Resistance pattern

	*st×1*	*st×2*	
1	+	−	TE
1	+	−	E
2	+	−	DOC, SXT, TE,
1	+	−	AML, AMP, CIP, E, SXT, TE
2	+	−	AML, AMP, CRO, DOC, E, SXT, TE
1	−	+	TE
1	−	+	AML, AMP, SXT, TE
1	−	+	AMP, CHL, E, SXT, TE
1	−	+	AML, AMP, CRO, DOC, E, SXT, TE

AML=Amoxicillin, AMP=Ampicillin, CRO=Ceftriaxone, CHL=Chloramphenicol, CIP=Ciprofloxacin, DOC=Doxycycline, E=Erythromycin, CN=Gentamicin, SXT=Trimethoprim/sulfamethoxazole, TE=Tetracycline, STEC=Shiga toxin-producing *Escherichia coli*

## Discussion

In the study area, the proportion of buffaloes carrying STEC was 11% (95% CI, 6.1-18.8%). Although the first report of STEC in slaughtered buffaloes dates back to 2008 [14], to the best of our knowledge based on exhaustive literature searches, there are no other reports from Bangladesh that predate our detection of the STEC in healthy buffaloes in Bangladesh. Previous studies in the same study area reported that 2% of the Black Bengal goat [11] and 5.4% of cattle [12] on smallholding were positive for sorbitol non-fermenting STEC, which suggest that, compared to cattle and goat, buffaloes have a higher probability of carrying STEC. This higher carriage of STEC in buffaloes in Bangladesh further supported by the study is conducted on slaughtered animals [14]. Islam *et al*. [14] reported a higher prevalence (38%) in the buffaloes sampled after slaughter. This variation in STEC carriage could be attributed to the status of the animal sampled (live or dead) and contamination during the slaughtering process. In the present study, the proportion of buffaloes carrying STEC was higher than the ranges reported from buffaloes in India (6.6%) [21], Italy (6.8%) [22] and Iran (7.2%) [23]. However, higher proportions of STEC carriage were reported in buffaloes from Brazil (64%) [9] and Vietnam (27%) [24]. The observation of this study, together with the finding by Islam *et al*. [14], indicated that probably the carriage proportion of STEC is widespread among buffaloes in Bangladesh.

In this study, 71% of fecal samples were positive for *E. coli*. This result might be due to two reasons: First, one possible reason is that some sampled animals might have been treated with antimicrobial before we collected the fecal samples from those animals. Second, up to 10% of *E. coli* isolates have historically been reported to be slow or non-lactose fermenting, which probably contribute to the outcome since we have considered only pink color colonies on MacConkey agar (lactose fermentation positive) and time was 24 h which might be not sufficient for the slow lactose-fermenting *E. coli*.

The distributions of Shiga toxin-producing genes in *E. coli* isolated in the present study indicated that the proportion of buffalo calves carrying STEC was lower than adult animals; however, the probability of carriage of STEC between calves and adult buffaloes did not differ significantly. This distribution STEC by age is corroborated by the previous investigation on goats [11] in the study area. This study recorded a significant difference (p=0.003) for the proportion of carriage of STEC in buffaloes with poor farm hygiene compared to buffaloes with good farm hygiene. This result of the present study implies good farm hygiene probably leading to the reduction of STEC carriage in animals [25]. We did not perform any risk factors analysis. Additional epidemiological investigations are required to identify the risk factors and their association with STEC carriage in animals.

Antimicrobial resistance profile of STEC can be a valuable indicator for assessing the status of emerging MDR STEC strains since we observed high MDR in STEC isolated from healthy buffaloes. We observed the highest antimicrobial resistance against TE (91% [10/11]) followed by SXT (73% [8/11]). This resistance profile is supported by the previous study on the caprine STEC isolated from the study area [26]. More than half of the STEC isolates (6/11) showed resistance against E and AMP. Johura *et al*. [27] reported a higher resistance against E (87%) in STEC isolated from livestock species in Bangladesh. A substantial proportion of STEC (73% [8/11]) isolated in the present study was resistant to more than one antimicrobial agent. The occurrence of MDR STEC in livestock species is corroborated by other studies [26,27]. These observations from the present study reflect the widespread and prolonged use of both antibiotics for the treatment of infectious diseases and in animal feeds as a growth promoter. Interestingly, 91% (10/11) STEC isolates were sensitive to CHL, CIP, and CN. This observation agrees with the study of Hamed *et al*. [28] who reported that CHL, CIP, and CN were sensitive to all STEC isolated from some food products and human stool in Egypt. These observations suggest CHL, CIP, and CN probably the choice of antibiotics in the control of STEC infection in animals and human; however, more clinical trials are required.

## Conclusion

This study is the first to confirm the occurrence of *E. coli* carrying Shiga toxin-producing genes in healthy buffaloes on smallholdings in Bangladesh. The proportion of buffaloes carrying STEC was about 11%. The high frequency of antimicrobial-resistant STEC found in buffaloes, together with other livestock species, probably poses added challenges to STEC infection control at the source in Bangladesh. Effort should be focused on the detection of resistance genes in STEC from animal and human cases to establish an effective MDR STEC surveillance in Bangladesh.

## Authors’ Contributions

MDG and AD conceived and designed the project. AS was involved in wet laboratory experimental works. MDG and AD carried out the organization of the data analysis and writing of the manuscript. All the authors read and approved the submitted version of the manuscript.
